# An In Silico Approach to Discover Efficient Natural Inhibitors to Tie Up Epstein–Barr Virus Infection

**DOI:** 10.3390/pathogens13110928

**Published:** 2024-10-25

**Authors:** Ayan Das, Mumtaza Mumu, Tanjilur Rahman, Md Abu Sayeed, Md Mazharul Islam, John I. Alawneh, Mohammad Mahmudul Hassan

**Affiliations:** 1 Department of Biochemistry and Molecular Biology, University of Chittagong, Chattogram 4331, Bangladesh; ayan.ae7788@gmail.com (A.D.); mumtazamumu@gmail.com (M.M.); tanjilur.cu@gmail.com (T.R.); 2 National Centre for Epidemiology and Population Health (NCEPH), College of Health and Medicine, The Australian National University, Canberra, ACT 2601, Australia; 3 Department of Animal Resources, Ministry of Municipality, Doha P.O. Box 35081, Qatar; walidbdvet@gmail.com; 4 Plant Biosecurity and Product Integrity, Biosecurity Queensland, Department of Agriculture and Fisheries, Brisbane, QLD 4000, Australia; john.alawneh@daf.qld.gov.au; 5 School of Veterinary Science, The University of Queensland, Gatton, QLD 4343, Australia; 6 Faculty of Veterinary Medicine, Chattogram Veterinary and Animal Sciences University, Chattogram 4225, Bangladesh

**Keywords:** Epstein–Barr virus, in silico screening, glycoprotein L, molecular docking, MD simulation

## Abstract

Epstein–Barr virus (EBV), also known as human herpesvirus 4, is a member of the herpes virus family. EBV is a widespread virus and causes infectious mononucleosis, which manifests with symptoms such as fever, fatigue, lymphadenopathy, splenomegaly, and hepatomegaly. Additionally, EBV is associated with different lymphocyte-associated non-malignant, premalignant, and malignant diseases. So far, no effective treatment or therapeutic drug is known for EBV-induced infections and diseases. This study investigated natural compounds that inhibit EBV glycoprotein L (gL) and block EBV fusion in host cells. We utilised computational approaches, including molecular docking, in silico ADMET analysis, and molecular dynamics simulation. We docked 628 natural compounds against gL and identified the four best compounds based on binding scores and pharmacokinetic properties. These four compounds, with PubChem CIDs 4835509 (CHx-HHPD-Ac), 2870247 (Cyh-GlcNAc), 21206004 (Hep-HHPD-Ac), and 51066638 (Und-GlcNAc), showed several interactions with EBV gL. However, molecular dynamics simulations indicated that the protein–ligand complexes of CID: 4835509 (CHx-HHPD-Ac) and CID: 2870247 (Cyh-GlcNAc) are more stable than those of the other two compounds. Therefore, CIDs 4835509 and 2870247 (Cyh-GlcNAc) may be potent natural inhibitors of EBV infection. These findings can open a new way for effective drug design against EBV and its associated infections and diseases.

## 1. Introduction

The historical Epstein–Barr virus (EBV) infection first arose in 1958 in Uganda. An English surgeon, Denis Burkitt, perceived frequent cancer among children in equatorial Africa, which was later named Burkitt’s lymphoma (BL) or Burkitt’s disease. Afterwards, Epstein, Achong, and Barr isolated herpes virus-like particles from infected tissue samples, and hence, the virus was named Epstein–Barr virus in 1964. Being the first human tumour virus, EBV infects approximately 90% of the adult population worldwide [1,2,3]. Individuals with healthy immunity might overcome the harmful effects of EBV infection, whereas immunosuppressive individuals develop several deadly cancers. Despite a notable impact on health, no well-established clinical management and treatment procedure is available for EBV infection. EBV infects two types of primary cells in the human body: the B lymphocytes and the epithelial cells [4]. Lymphatic diseases like infectious mononucleosis, Hodgkin’s disease (HD), Burkitt’s lymphoma (BL), post-transplant lymphoproliferative disorders (PTLDs), T-cell lymphomas, and epithelial diseases such as oral hairy leukoplakia (OHL), nasopharyngeal carcinoma (NPC), and undifferentiated gastric carcinoma constitute the whole EBV infection [5,6]. Often, being an orally transmitted virus, EBV spreads through saliva or contact with the airborne virus, blood transfusion, sexual contact, and tissue transplantation [7,8,9]. EBV infection is often asymptomatic, although infectious mononucleosis is the prototypic form of the disease, including symptoms like fever, sore throat, cervical and generalised lymphadenopathy, hepatosplenomegaly, and somatic complaints of fatigue and malaise [10,11].

The Epstein–Barr virus, also known as Human gammaherpesvirus 4, is one of the nine known human herpesvirus types in the herpes family. It belongs to the order *Herpesvirales*, family *herpesviridae***,** subfamily *gammaherpesvirinae,* and the genus *lymphocytovirus* [12]. EBV measures from 120 to 180 nm along with a double-stranded linear DNA (~171 Kb long), encoding ~90 genes [13]. An envelope surrounding the viral genome, protein nucleocapsid, and viral tegument, and containing both lipids and surface projections is made of glycoproteins and crucial for host cell infection [10]. Two significant strains with high similarities, type 1 and type 2, are reported with several variants or sub-types under each section. EBV-1 and EBV-2 can be distinguished based on the four latent genes (*EBNA2*, *EBNA3A*, *EBNA3B*, and *EBNA3C*) [14,15]. To induce in vitro growth transformation, EBV-1 acts more actively than EBV-2, but in both cases, the infection can be lifelong without any type-specific characteristics [16]. Among the 13 glycoproteins encoded by the EBV genome, 12 are essential to the lytic replication cycle, and the resting one may be crucial to latency. Eleven proteins constitute the virion envelope; the other two are nonstructural proteins. The viral glycoproteins can be categorised into three groups: proteins that help in virus entry and spread, proteins involved in virus assembly, and proteins involved in manipulating the host cell. Each of the proteins can contribute multiple functions to viral pathogenesis [17].

Several anti-EBV neutralising monoclonal antibodies (mAbs) have been designed in previous works targeting several glycoproteins, including glycoprotein H (gH), glycoprotein L (gL), glycoprotein B, glycoprotein 42 (gp42), and glycoprotein 350 (gp350). Almost all the antibodies failed to inhibit B cell or epithelial cell infection. The 72A1 mAb, C1 mAb (anti-gp350), and F-2-1 (anti-gp42 mAb) potently block the B cell infection but fail to neutralise infection of epithelial cells [18,19,20,21,22,23,24,25,26]. The anti-gHgL antibodies, E1D1, CL59, and CL40, neutralise epithelial cell infection but fail to block B cell infection [19,21,24,26,27]. Only the AMMO1, an anti-gHgL antibody that binds to D-I elements (including gL), D-II, and the linker helix of the gHgL complex, potently blocks both the B cell and epithelial cell infection in the host body [28]. However, further clinical trials and observations are needed to ensure the safety, efficiency, and side effects of this newly developed vaccine.

Fusion mechanisms of EBV in both B cells and epithelial cells require the contribution of heterodimer gHgL complex [29]. The GHG complex of EBV forms a high-affinity contact with gp42, mediating entry into the B cells. The complex also directly triggers the epithelial cells’ entry through integrin receptors [19]. Moreover, this complex also regulates gB activation, mediating membrane fusion through conformational change [30,31,32]. Mutations around the D-I and D-I/D-II interfaces of the gHgL complex have the most negative influence on fusion mechanisms, as conformational changes of these domain regions may trigger the membrane fusion of EBV [33,34]. Both the gp42 and gHgL complexes act as active regulators of overall fusion processes, implying more importance on their active involvement in fusion [35,36]. Prior studies suggested a possible role of gL in the folding pattern of gH and its transportation to the cell membrane. gL may also play a crucial role in the binding of gH to gp42 in the fusion mechanism [24,37,38]. Inhibition of gL or any conformational change may inactivate the gHgL complex and the whole fusogenic mechanism. So, in this work, we have evaluated the gL as the significant target protein to filter out some efficient natural inhibitors through computational methods.

Besides many other options to solve EBV infection, in silico approaches can be a good option. In silico drug design is now an intense field that evaluates different compounds against specific pathogens. Computer-aided drug designing (CADD) methods are cost-effective and time-saving approaches that make the drug-developing stages easier and more facile [39]. This study used in silico techniques to evaluate different natural compounds against the EBV pathogenic protein, envelope gL. Natural compounds are significant in clinical approaches against viral or bacterial infections. Nearly one-third of the popular drugs worldwide are developed from natural compounds or their derivatives. Various structural configurations, pharmacological activities, and favourable pharmacokinetics biassed our compound selection to natural compounds rather than synthetic molecules [40,41,42]. Natural compounds include larger reactive fragments in contrast to synthetic drugs [43]. Hence, we have chosen several natural compounds as candidate inhibitors in this study to analyse their inhibitory mechanism against the EBV pathogenic protein gL through in silico approaches.

## 2. Materials and Methods

### 2.1. Protein Preparation and Control Ligand Selection

The three-dimensional (3D) crystal structure of EBV gL with a resolution of 4.80 Å was retrieved from the RCSB Protein Data Bank (PDB) with PDB ID 6C5V [28,44]. Since only gL was our target protein against which docking of candidate compounds needed to occur, we removed the glycoprotein H, glycoprotein 42, other protein chains, protein cofactors, water molecules, and metal ions. The protein cofactors and metal ions were removed to skip unwanted interference during docking simulations [45]. Subsequently, we added polar hydrogen atoms and merged non-polar hydrogen atoms using BIOVIA Discovery Studio Visualizer v21.1.0.20298 [46]. Gasteiger charges were calculated using AutoDock tools v4.2.6 [47]. Finally, the control ligand, NAG (2-acetamido-2-deoxy-beta-D-glucopyranose), associated with gL was downloaded from the PubChem database with PubChem CID: 24139 [48].

### 2.2. Compound Retrieval and Preparation

As natural compounds are highly known for their unique scaffolds, vast diversity, and better solubility, we opted to screen a library of natural compounds for our procedure. Ambinter (http://www.ambinter.com/; accessed on 10 August 2024) is a French company with an enriched database including nearly 36 million compound structures, which can be searched based on similarity to a reference structure or through Simplified Molecular Input Line Entry System (SMILES) or International Union of Pure and Applied Chemistry (IUPAC) chemical names. This database is well known for searching for natural compounds, and we can also order any compounds for further in vivo or in vitro analysis from their website. While it is practically impossible to screen millions of compounds, it is also true that compounds with high structural similarity and minor but efficient diversities to the control ligand of a protein show promising possibilities of working as an inhibitor. Compounds with highly diverse structures to the control ligand are unlikely to show proper binding affinity. Therefore, we searched for the Ambinter natural compound library and selected compounds that showed 90% structural similarity to the control ligand (PubChem CID: 24139) attached to gL. The compounds considered for further virtual screening were natural and in stock. We used Open Babel v2.4.0. and AutoDock v4.2.6 to prepare the retrieved compounds and the control ligand. These tools were employed to merge non-polar hydrogen atoms, detect aromatic carbons, and set the ‘torsion tree’ [47,49].

### 2.3. Active Site Prediction

We used the CASTp 3.0 program [50] to identify binding pockets throughout the protein, keeping the radius probe at the default value of 1.4 Å. This programme employs computational geometry algorithms, including Delaunay triangulation, alpha shape, and discrete flow. The server filtered the computed cavities using Richards’ and Connolly’s surfaces and shortened them by decreasing volume and surface area. We also used the FTMap server [51] to identify the optimal binding pocket on gL. The FTMap server placed small probes of organic molecules (varying in size, shape, and polarity) on the protein’s molecular surface. By evaluating each probe’s most favourable position and clustering them, the server calculated the average energy and ranked the clusters. It then displayed a contact graph showing the contact frequency per amino acid for all non-bonded and hydrogen-bonded interactions.

### 2.4. Molecular Docking

The site-specific molecular docking analysis was conducted using the PyRx v0.9.2 virtual screening tool AutoDock v2.4.6 [47]. We focused on pocket 2, as identified by CASTp 3.0 and the FTMap server (accessed on 10 August 2024), to determine the binding mode of the desired protein with selected natural compounds. This pocket also included the highly interactive amino acids, including those utilised by the control ligand, whereas no other predicted pockets covered them. PyRx v0.9.2 is a free virtual screening tool widely used for molecular docking, data preparation, and analysis, and it supports all major operating systems (OS), including Linux, Windows, and Mac OS. PyRx v0.9.2 provides AutoDock and AutoDock Vina as docking wizards with an intuitive user interface, making it an efficient computer-aided drug design (CADD) tool. Pocket 2 of the protein, defining the assumed active site, was covered by a grid box with specific coordinates and parameters, as shown in Table 1. After the docking procedure, using the default configuration parameters of PyRx v0.9.2, several compounds with the top binding energies (kcal/mol) showing negative values were selected for further evaluation. Finally, the binding interactions of the protein–ligand complexes were observed using the BIOVIA Discovery Studio Visualizer v21.1.0.20298 [46].

### 2.5. Pharmacokinetics Properties Prediction

Designing and developing a novel drug includes the analysis of pharmacokinetic (pk) properties to ensure the drug’s safe and balanced behaviour inside the human body. The best compounds (based on docking scores) were evaluated using multiple pharmacokinetic criteria, including absorption, distribution, metabolism, and excretion (ADME) [52]. These critical parameters help in filtering novel drugs or inhibitors for specific targets [52]. We used the SwissADME server (http://www.swissadme.ch; accessed on 10 August 2024) [53], to assess our selected compounds based on different parameters, including molecular weight (g/mol), number of heavy atoms, number of aromatic heavy atoms, number of rotatable bonds, number of H-bond acceptors and donors, LogP, LogS, level of gastrointestinal (GI) absorption, blood–brain barrier (BBB) permeability, synthetic accessibility, and the number of Lipiniski’s violation [54]. Additionally, we employed the pkCSM server (http://biosig.unimelb.edu.au/pkcsm/; accessed on 10 August 2024) [55], to examine the Caco2 permeability, P-glycoprotein (P-gp) substrate, and inhibitor predictions.

### 2.6. Toxicity Prediction

Taking medicines with varying levels of toxicity can lead to severe complications for humans and other animals. Toxicity analysis measures the degree to which chemical compounds can be toxic and cause organ damage. Predicting toxicity is valuable in filtering out potentially harmful compounds, which can be further evaluated in clinical trials. Thus, toxicity observation is a crucial aspect of the computer-aided drug design (CADD) process. 

We used the pkCSM web-based server (http://biosig.unimelb.edu.au/pkcsm/; accessed on 10 August 2024) [55] to evaluate the toxicity of our chosen compounds, considering AMES toxicity, hepatotoxicity, human Ether-à-go-go-Related Gene (hERG) inhibition, oral rat acute toxicity (LD50), oral rat chronic toxicity (LOAEL), skin sensitisation, *Tetrahymena pyriformis* toxicity, and minnow toxicity. Additionally, we employed the admetSAR 2.0 online server (http://lmmd.ecust.edu.cn/admetsar2; accessed on 10 August 2024) [56] to analyse our selected compounds’ carcinogenicity, androgen receptor (AR) binding, honey bee toxicity, and fish aquatic toxicity analysis.

### 2.7. Molecular Dynamics Simulation

Molecular dynamics (MD) is crucial for predicting the dynamic behaviour and structural stability of protein–ligand complexes in a particular physiological environment, which is essential in drug discovery. This method analyses small molecules’ strength and binding interactions with the target receptor, examining the protein–ligand complex for interactions and flexibility. 

We used the Desmond package within the Schrödinger suite v2024-3 to perform a 100 ns MD simulation for the complexes formed between the selected four compounds, the control ligand, and the target protein [57]. The 100 ns MD simulation length has been sufficient for observing stable interactions and key features involving EBV proteins in other studies [45,58]. Moreover, we found that the complex formed between our ligands and target protein generally stabilised after approximately 80 ns of simulation in several exploratory simulations. Before the simulation, the protein–ligand complexes were prepared using the protein preparation wizard [59]. An orthorhombic boundary box with dimensions of 10 × 10 × 10 Å^3^ was allocated for each complex with a simple point charge (SPC) water model. A 0.15 M salt concentration was maintained by randomly distributing Na^+^ and Cl^−^ ions throughout the solvated system. Additionally, the system was relaxed and minimised using the OPLS3e force field [60]. 

The constant pressure-constant temperature (NPT) ensemble was maintained with a constant temperature of 300.0 K along with a fixed pressure of 1.01325 bar [61,62]. Each complex was first allowed to relax, and then an energy level of 1.2 kcal/mol was applied during the 100 ps analysis [63]. Applying a 1.2 kcal/mol threshold at each 100 ps interval is crucial for identifying stable and significant interactions above the energy threshold. After the production phase, various dynamic analyses were performed, including root mean square deviation (RMSD), root mean square fluctuation (RMSF), the radius of gyration (rGyr), solvent accessible surface area (SASA), and protein–ligand interactions to evaluate the stability and flexibility of the protein–ligand complexes.

## 3. Results

**Figure 1:**
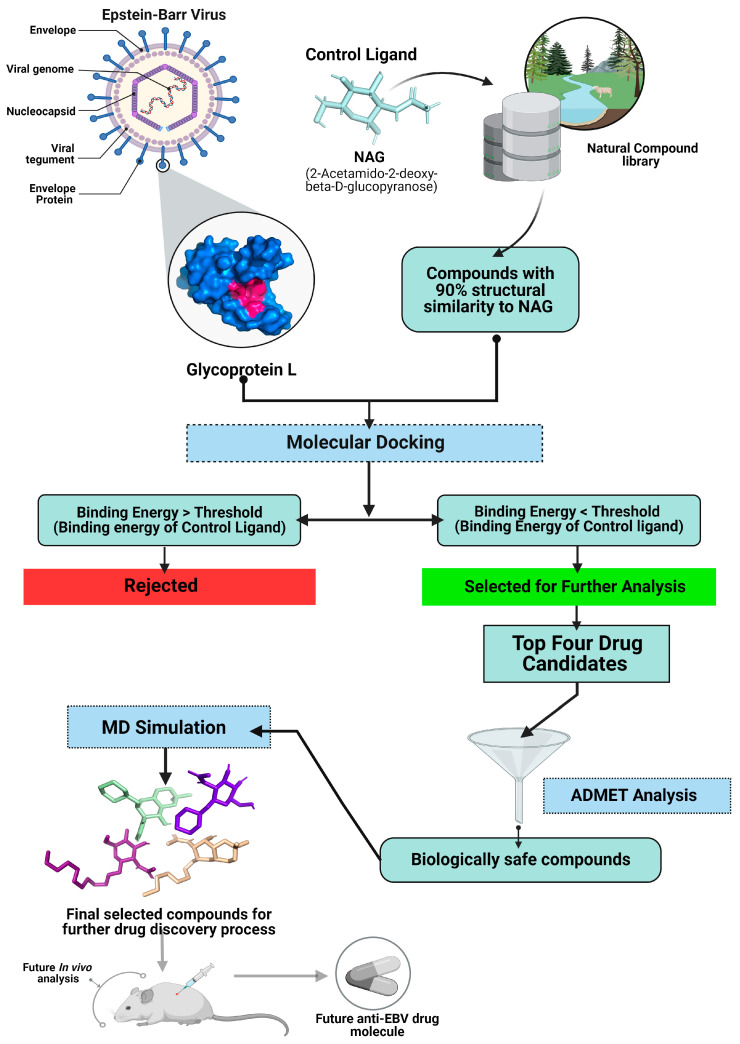
The illustration of structure-based in silico screening processes for identification of potential drug candidates against glycoprotein L. The workflow includes retrieving and preparing protein and drug candidate compounds, molecular docking, ADMET analysis, and molecular dynamic simulation processes. Further in vivo processes may aid in evaluating new efficient drugs against EBV as shown in the faded diagrams in this figure.

The necessity of EBV gL in virus fusion and disease generation has already been proven [24,28,29,37]. Disturbing the general conformation of gL using different natural compounds may inhibit the EBV fusion mechanisms and viral infections. For structure-based drug design, we applied virtual screening, molecular docking studies, ADMET analysis, and molecular dynamic simulation, as shown in Figure 1, to identify novel and potential inhibitors against EBV and to explore a new treatment option for the associated diseases.

### 3.1. Natural Compounds Exploration and Retrieval

We selected natural compounds as drug candidates due to their disease-inhibiting properties and high chemical diversity. Additionally, they are less toxic and more effective [64]. Natural compound libraries typically consist of 60–65% plant-derived compounds, 5–10% isolated from microorganisms, about 5% from marine species, and the rest from other natural sources [65]. 

In our study, we employed similarity-based virtual screening and collected compounds from the Ambinter natural library. We identified a total of 628 natural compounds with 90% structural similarity to the control ligand. All of these compounds are available for purchase from this server for further in vivo analysis. Ambinter provides structural information on compounds in both SDF and CSV formats. The Pyrx Virtual Screening tool was used to prepare the retrieved compounds. Open Babel utilised the universal force field (UFF) [49] for energy minimisation and converted the compounds to the PDBQT format, which is capable of docking using AutoDock Vina [47].

### 3.2. Active Site Prediction

**Figure 2:**
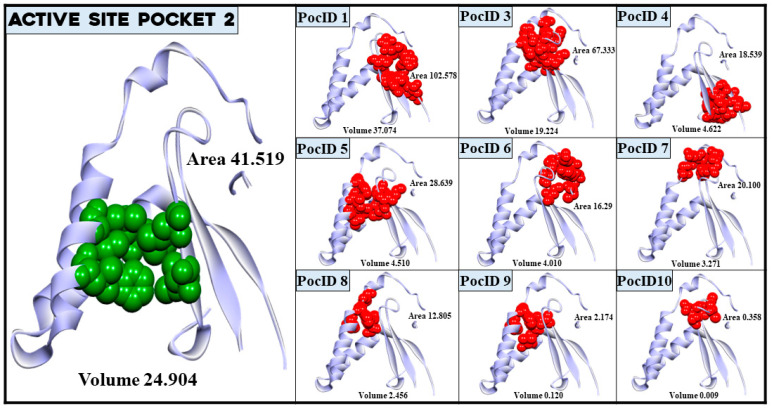
The visualisation of the assumed active site pocket and other binding sites in glycoprotein L with their area (Å^2^) and volume (Å^3^). The green-coloured balls represent amino acids included in our assumed active site, the PocID 2, as identified by the CASTp 3.0 server. The red-coloured balls show amino acids of other pockets (PocID 1, PocID 3, PocID 4, PocID 5, PocID 6, PocID 7, PocID 8, PocID 9, and PocID 10) with their position in the gL protein, respectively.

Using the CASTp 3.0 program, we visualised our target protein’s top ten probable binding pockets, gL, as depicted in Figure 2 and Supplementary Table S1 [50]. We used the FTmap server to identify the top 10 amino acids contributing to hydrogen bonds and non-bonded interactions (Supplementary Table S2) to predict the most suitable binding site [66]. Among the seven amino acids in pocket 2, as suggested by CASTp 3.0 and shown in Table 2, four are among the top 10 residues identified by the FTmap server. Additionally, pocket 2 was the second largest and third largest pocket in terms of surface-accessible surface volume and area, respectively (Volume 24.904 Å^3^ 41.519 Å^2^) (Figure 2). Therefore, pocket 2 is supposed to be the active site on our target protein, gL.

### 3.3. Molecular Docking Analysis

Molecular docking is the most widely used method in structure-based drug design to analyse the binding affinity of specific small molecules with targeted protein binding sites and estimate the detailed molecular basis of interactions [67]. We performed molecular docking in a grid box (Table 1), which was set to cover the assumed active site of gL (pocket 2) and its adjacent residues. Possible drug candidates usually contain higher binding energy with a negative sign than the natural ligand associated with a protein molecule. To ensure that the virtual screening will end up only with highly bound compounds to our target protein, we determined a threshold equal to the binding affinity of the control ligand. A total of 398 candidate compounds among the total 628 were successful at passing through our selection criteria of binding affinity (−3.9 kcal/mol). We chose the best four compounds based on the binding affinity for further analysis. Among the selected four compounds, the compound N-(6-Cyclohexyloxy-8-hydroxy-2,2-dimethyl-4,4a,6,7,8,8a-hexahydropyrano[3,2-d][1,3]dioxin-7-yl)acetamide (CHx-HHPD-Ac) with PubChem CID: 4835509 had the lowest docking score of −5.8 kcal/mol. Additionally, the compounds Cyclohexyl 2-(acetylamino)-2-deoxyhexopyranoside (Cyh-GlcNAc), N-[(4Ar,6R,7R,8R,8aS)-6-heptoxy-8-hydroxy-2,2-dimethyl-4,4a,6,7,8,8a-hexahydropyrano[3,2-d][1,3]dioxin-7-yl]acetamide (Hep-HHPD-Ac) and Undecyl 2-acetamido-2-deoxy-B-D-glucopyranoside (Und-GlcNAc) with PubChem CIDs: 2870247, 21206004, and 51066638, respectively, exhibited docking scores of −5.7 kcal/mol, −5.6 kcal/mol, and −5.5 kcal/mol, respectively (Table 3).

### 3.4. Binding Pattern Analysis of Selected Molecules

**Figure 3:**
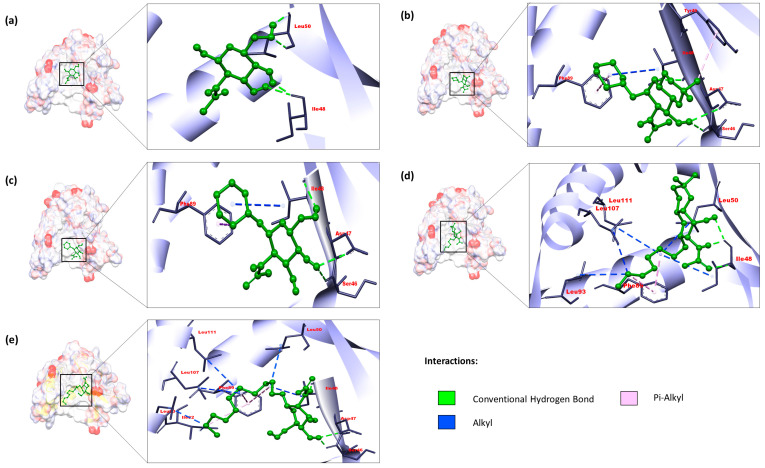
The 3D-interaction diagrams of protein–ligand complexes derived from the control ligand and the best four compounds according to the docking scores. All the diagrams were generated using the BIOVIA Discovery Studio Visualizer tool. The upper left section (**a**) shows the interaction of the control ligand PubChem CID: 24139 (NAG). The upper right section (**b**), middle left section (**c**), middle right section (**d**), and lower left section (**e**) correspond to the compounds PubChem CID: 4835509 (CHx-HHPD-Ac), PubChem CID: 2870247 (Cyh-GlcNAc), PubChem CID: 21206004 (Hep-HHPD-Ac), and PubChem CID: 51066638 (Und-GlcNAc), respectively.

**Figure 4:**
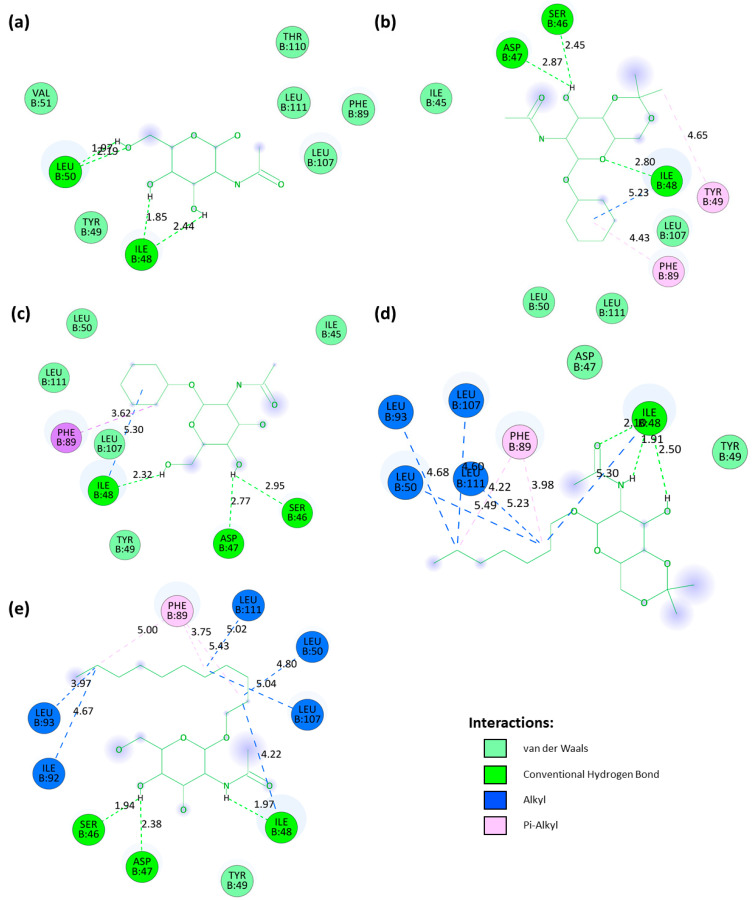
The 2D-interaction diagrams of protein–ligand complexes with bond distances derived from the best four compounds according to the docking scores. All the diagrams were generated using the BIOVIA Discovery Studio Visualizer tool. The upper left section (**a**) shows the interaction of the control ligand PubChem CID: 24139 (NAG). The upper right section (**b**), middle left section (**c**), middle right section (**d**), and lower left section (**e**) correspond to the compounds PubChem CID: 4835509 (CHx-HHPD-Ac), PubChem CID: 2870247 (Cyh-GlcNAc), PubChem CID: 21206004 (Hep-HHPD-Ac), and PubChem CID: 51066638 (Und-GlcNAc), respectively.

All four drug candidates were bound firmly with gL. Their binding interactions were observed using the BIOVIA Discovery Studio Visualizer v21.1.0.20298 and elaborated in Table 4. The control ligand of gL (PubChem CID: 24139) formed two H-bonds with ILE48 and two with LEU50. Based on the binding affinity, our top candidate compound, PubChem CID: 4835509 (CHx-HHPD-Ac), was found to form conventional hydrogen bonds and hydrophobic bonds. It formed conventional H-bonds with residues SER46, ILE48, and ASP47. It interacted with ILE48 and PHE89 residue by forming hydrophobic bonds. The compound PubChem CID: 2870247 (Cyh-GlcNAc) also interacted with these four amino acid residues (SER46, ASP47, ILE48, and PHE89). The compound with PubChem CID: 21206004 (Hep-HHPD-Ac) was observed to interact with ILE48 residue by forming three conventional H-bonds and one alkyl hydrophobic bond through different atoms. This compound also formed hydrophobic bonds with PHE89, LEU93, LEU107, and LEU111. The PubChem CID: 51066638 (Und-GlcNAc) compound contained maximum bonds with the target protein. Interactions between this compound and gL were established via conventional H bonds involving amino acids SER46, ASP47, and ILE48 and hydrophobic bonds involving ILE48, LEU50, PHE89, LEU93, LEU107, and LEU111 residues. After interaction analysis, we found that eight amino acid residues of the gL interacted with the top four drug candidates. These interacting residues included amino acids SER46, ASP47, ILE48, LEU50, PHE89, LEU93, LEU107, and LEU111 (Table 4). ILE48, LEU50, PHE89, LEU107, and LEU111 constituted the assumed active site for gL (Table 2). The receptor-ligand interactions were depicted with bond distances through 3D and 2D visualisations (Figure 3 and Figure 4).

### 3.5. ADME Analysis

Drug pharmacokinetics describes the interaction between drugs and the human body, such as absorption, distribution, metabolism, and excretion (ADME). We observed physicochemical (PC) properties, lipophilicity, solubility, GI absorption, BBB permeant, synthetic accessibility, drug-likeness, and other characteristics for ADME profile analysis. ADME includes consideration of Lipinki’s rules of five, which is a significant requirement for evaluating an orally active drug [69]. We followed all ADME profile analysis rules and found the best four compounds showing no violation of Lipinski’s rules of five. The results of the ADME analysis for all four selected compounds are represented in Table 5. As they obeyed Lipinski’s rules of five, their LogP values were below 5. Compounds with PubChem CID: 4835509 (CHx-HHPD-Ac), PubChem CID: 21206004 (Hep-HHPD-Ac), and PubChem CID: 51066638 (Und-GlcNAc) had positive LogP values, meaning they have higher affinities for the lipid phase. The compound with PubChem CID: 2870247 (Cyh-GlcNAc) had a negative LogP value and showed more solubility in the aqueous phase. Their LogP values also influenced their permeability through membranes, potency, selectivity, and promiscuity. Our top four compounds contained Log S (ESOL) values ranging from −3.18 to −0.99, indicating that they are soluble or highly soluble in water and can easily be absorbed by our bodies. They showed high GI absorption and couldn’t cross the BBB as described by the ADME result.

### 3.6. Toxicity Analysis

In silico toxicity, measurement is essential for better potential drug candidate selection before undergoing a clinical trial. Drug toxicity analysis helps to determine the level of damage that a compound can cause to an organism or substructures of the organism, such as cells and organs. Our analyses of several toxicological parameters, such as AMES toxicity, oral rat acute toxicity (LD50), oral rat chronic toxicity (LOAEL), hepatotoxicity, etc., for the top four drug candidates are shown in Table 6. All of them showed negative results on the AMES test. Thus, they can’t cause mutations in the DNA of the test organism. Although a diverse range of drug structures can cause hepatotoxicity, our selected drug candidates are not hepatotoxic, which means they will not harm human livers. Furthermore, they will not express any allergic response to the skin because their skin sensitisation results were also negative.

### 3.7. Molecular Dynamics (MD) Simulation

The stability and flexibility of a protein–ligand complex in a particular environment for a fixed period can be understood and analysed using molecular dynamics (MD) simulation. In this study, the four best compounds (CID: 4835509 (CHx-HHPD-Ac), CID: 2870247 (Cyh-GlcNAc), CID: 21206004 (Hep-HHPD-Ac), and CID: 51066638 (Und-GlcNAc)) based on their binding affinity against the target protein, gL, were subjected to MD simulation alongside the control (CID: 24139). The conformational changes of the gL protein in each protein–ligand complex were investigated by deploying a 100 ns MD simulation. Several parameters of the MD simulation, such as RMSD, RMSF, SASA, rGyr, and protein–ligand interactions, were used in this study to measure and analyse the stability of the complexes. 

### 3.8. Analysis of RMSD

**Figure 5:**
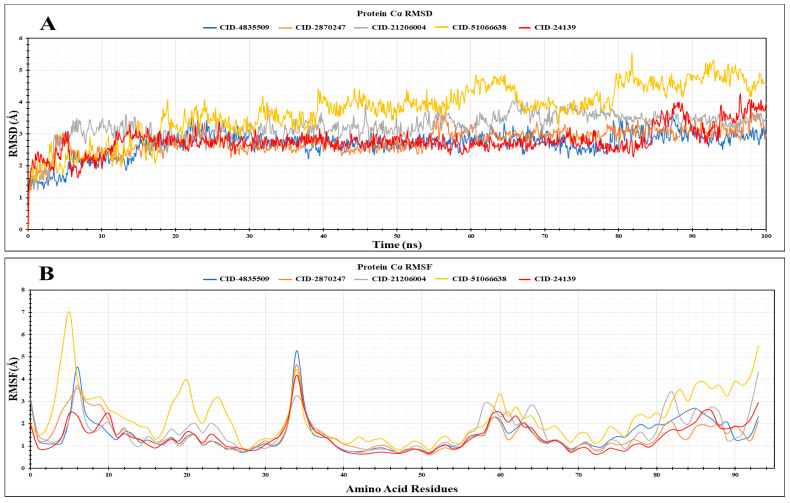
(**A**) The RMSD values of the gL protein of EBV bound with four selected compounds and control are given. (**B**) The RMSF values of the residues of the gL proteins binding upon four selected ligands and control are shown here. The RMSD and RMSF values for the CID: 4835509 (CHx-HHPD-Ac), CID: 2870247 (Cyh-GlcNAc), CID: 21206004 (Hep-HHPD-Ac), CID: 51066638 (Und-GlcNAc), and CID: 24139 (control) are present in blue, orange, grey, yellow, and red colours, respectively.

The deviation of a protein’s structure compared to its initial position in a protein–ligand complex can be measured by root mean standard deviation. It is an essential parameter in the MD simulation that can give an idea of the durability and conformational changes in the protein’s core structure during the simulation time [70,71]. Figure 5A demonstrates the RMSD values of the complexes of the best four ligands and the control ligand. The average RMSD values for CID: 4835509 (CHx-HHPD-Ac), CID: 2870247 (Cyh-GlcNAc), CID: 21206004 (Hep-HHPD-Ac), and CID: 51066638 (Und-GlcNAc) were 2.68 Å, 2.78 Å, 3.23 Å, and 3.73 Å, respectively. Additionally, the average RMSD for the complex of the control ligand is 2.79 Å. The average RMSD values of all compounds fell between 2.5 Å and 3.8 Å. The complexes of CID: 4835509 (CHx-HHPD-Ac) and CID: 2870247 (Cyh-GlcNAc) showed more durability during the simulation period compared to the control, whereas the stability of the other two complexes was lacking. The complex of the CID: 51066638 (Und-GlcNAc) exhibited much fluctuation between 50 and 100 ns during the simulation period. The highest RMSD values for the complexes of the CID: 4835509 (CHx-HHPD-Ac), CID: 2870247 (Cyh-GlcNAc), CID: 21206004 (Hep-HHPD-Ac), and CID: 51066638 (Und-GlcNAc) were 3.65 Å, 3.74 Å, 4.24 Å, and 5.54 Å, respectively, whereas the lowest values were 1.22 Å, 1.18 Å, 1.14 Å, and 1.16 Å, respectively. Likewise, the highest and lowest RMSD values for the complex of control were 4.26 Å and 1.21 Å, respectively.

### 3.9. Analysis of RMSF

The interactions between a ligand and a protein’s specific amino acids (AA) in a protein–ligand complex could cause fluctuations in the protein’s backbone. These fluctuations can be measured by a parameter called root, which means standard fluctuation during MD simulation. We calculated the RMSF values of the complexes of the four best compounds and the control ligand to evaluate the gL’s structural flexibility upon binding with different ligands compared to its binding with the control ligand. The average RMSF values for CID: 4835509 (CHx-HHPD-Ac), CID: 2870247 (Cyh-GlcNAc), CID: 21206004 (Hep-HHPD-Ac), CID: 51066638 (Und-GlcNAc), and the control were 1.50 Å, 1.48 Å, 1.64 Å, 2.26 Å, and 1.41 Å, respectively. Figure 5B represents the RMSF values of the gL for the four best compounds and the control ligand. In Figure 5B, it is shown that there are lots of fluctuations at the protein’s beginning and end for all ligands, including the control. The reason behind this is the existence of the N- and C-terminals in the protein structure. Additionally, the protein showed fluctuations for all the ligands and the control between 30 and 40 AA residues. However, CID: 4835509 (CHx-HHPD-Ac) showed the highest fluctuation in this region. Furthermore, slight fluctuations were observed between 55 and 65 AA residues of the protein. After analysis of all RMSF for all complexes, it was found that CID: 51066638 (Und-GlcNAc) was less stable than other ligands, including control. Moreover, the protein showed additional fluctuations between 17 and 27 AA residues for CID: 51066638 (Und-GlcNAc). The highest RMSF values for the complexes of the CID: 4835509 (CHx-HHPD-Ac), CID: 2870247 (Cyh-GlcNAc), CID: 21206004 (Hep-HHPD-Ac), and CID: 51066638 (Und-GlcNAc) were 5.26 Å, 4.65 Å, 4.34 Å, and 7.01 Å, respectively, whereas the lowest RMSF values for these ligands were 0.70 Å, 0.60 Å, 0.68 Å, and 0.83 Å, respectively. Likewise, the highest and lowest RMSF values for the complex of control (CID: 24139) were 4.19 Å and 0.64 Å, respectively.

### 3.10. Radius of Gyration (rGyr)

**Figure 6:**
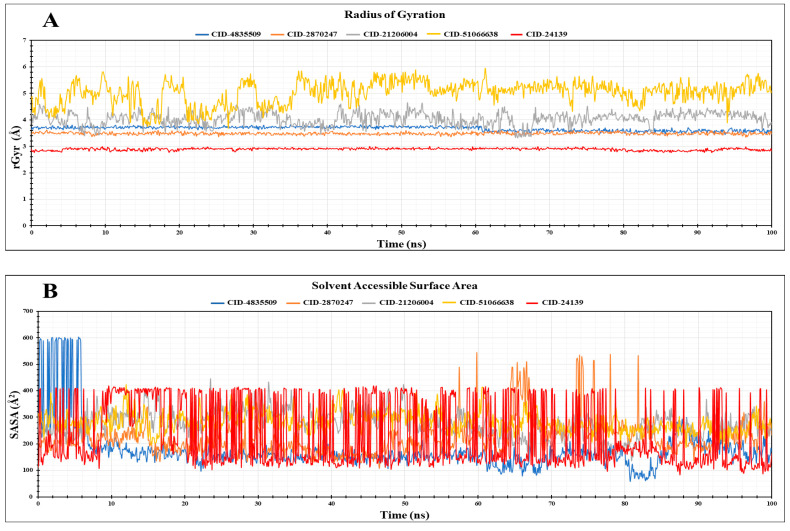
(**A**) Representing the radius of gyration (rGyr) of the five complexes for the four selected molecules and control obtained from the MD simulation. (**B**) The SASA values of the target protein bound with four selected ligands, as well as the control ligand are shown. Both rGyr and SASA values for the CID: 4835509 (CHx-HHPD-Ac), CID: 2870247 (Cyh-GlcNAc), CID: 21206004 (Hep-HHPD-Ac), CID: 51066638 (Und-GlcNAc), and CID: 24139 (control) are present in blue, orange, gray, yellow, and red colour, respectively.

The radius of gyration, or rGyr, refers to the atomic rearrangement of a protein–ligand complex about its axis. This is another important parameter used to predict the functionality and compactness of the protein upon binding with a ligand. We measured the rGyr values of the complexes of CID: 4835509 (CHx-HHPD-Ac), CID: 2870247 (Cyh-GlcNAc), CID: 21206004 (Hep-HHPD-Ac), and CID: 51066638 (Und-GlcNAc) to find out the compactness of the gL upon binding with our four best compounds (Figure 6A). We also calculated the rGyr for the control ligand complex to compare the results of the selected compounds. The average rGyr values for CID: 4835509 (CHx-HHPD-Ac), CID: 2870247 (Cyh-GlcNAc), CID: 21206004 (Hep-HHPD-Ac), and CID: 51066638 (Und-GlcNAc), and the control were 3.66 Å, 3.49 Å, 4.04 Å, 5.02 Å, and 2.89 Å, respectively. It is shown in Figure 6A that the first two compounds, CID: 4835509 (CHx-HHPD-Ac) and CID: 2870247 (Cyh-GlcNAc), have much better results than the other two compounds, such as CID: 21206004 (Hep-HHPD-Ac) and CID: 51066638 (Und-GlcNAc), compared to the control ligand. Among the four complexes for four selected compounds, the protein–ligand complex for CID: 2870247 (Cyh-GlcNAc) demonstrated less fluctuation in their rGyr during the 100 ns MD simulation. 

### 3.11. Solvent Accessible Surface Area (SASA)

Solvent accessible surface area (SASA) is the area of the protein in a protein–ligand complex that a solvent can access. We exploited this parameter of MD simulation to determine the accessible area of our target protein when bound with the four compounds or the control. This will indicate the stability and behaviour of the protein in the complex in different solvents, like hydrophobic or hydrophilic. Moreover, this will also help us to understand how the protein and its ligand will work together in the presence of solvents. The SASA values for the CID: 4835509 (CHx-HHPD-Ac), CID: 2870247 (Cyh-GlcNAc), CID: 21206004 (Hep-HHPD-Ac), CID: 51066638 (Und-GlcNAc), and control are presented in Figure 6B. The average SASA for CID: 4835509 (CHx-HHPD-Ac), CID: 2870247 (Cyh-GlcNAc), CID: 21206004 (Hep-HHPD-Ac), and CID: 51066638 (Und-GlcNAc) were 177.14 Å2, 221.95 Å2, 276.78 Å2, and 282.96 Å2, respectively. The average SASA for the control was 232.04 Å2. Therefore, it was found that the protein gL had less solvent-accessible area when bound with the first two compounds, CID: 4835509 (CHx-HHPD-Ac) and CID: 2870247 (Cyh-GlcNAc), compared to the control ligand’s result. This indicates the strong binding of the CID: 4835509 (CHx-HHPD-Ac) and CID: 2870247 (Cyh-GlcNAc) with our target protein. However, the other two compounds had less favourable results than those mentioned.

### 3.12. Protein–Ligand Contact Analysis

**Figure 7:**
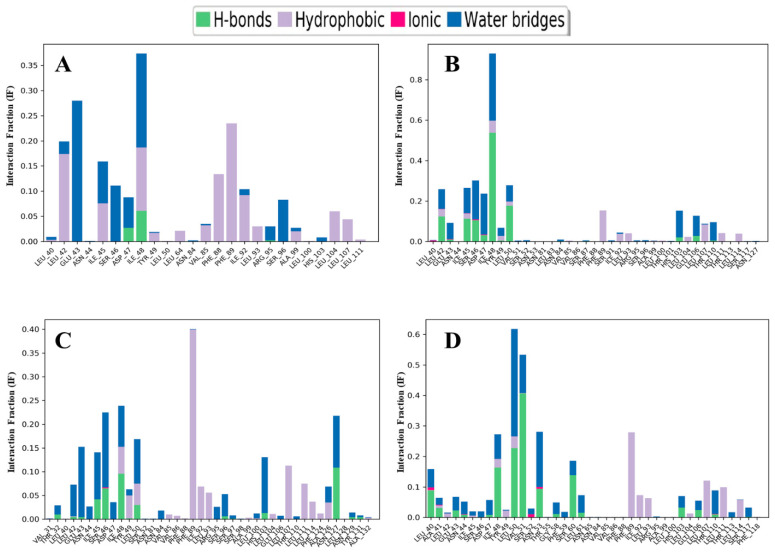
The interactions of (**A**) CID: 4835509 (CHx-HHPD-Ac), (**B**) CID: 2870247 (Cyh-GlcNAc), (**C**) CID: 21206004 (Hep-HHPD-Ac), and (**D**) CID: 51066638 (Und-GlcNAc) with the target protein gL are given.

A simulation interaction diagram (SID) was utilised to evaluate the protein–ligand interactions and understand the architecture of the complexes of the four compounds with the protein gL during the 100 ns MD simulation (Figure 7). The protein and the ligands involved several different bonds, such as hydrogen bonds, water bridges, ionic bonds, non-covalent bonds, etc., in their complexes. The compound CID: 4835509 (CHx-HHPD-Ac) formed multiple bonds with the gL at ASP47 and ILE48 residues with interaction fraction (IF) values of 0.38 and 0.08, respectively (Figure 7A). On the other hand, the compound CID: 2870247(Cyh-GlcNAc) interacted with the protein by forming multiple bonds at LEU42, GLU43, ILE45, SER46, ASP47, ILE48, TYR49, and LEU50 residues with the IF values of 0.25, 0.1, 0.25, 0.3, 0.25, 0.9, 0.05, and 0.3, respectively (Figure 7B). Likewise, the compound CID: 21206004 (Hep-HHPD-Ac) showed multiple interactions with gL at LEU42, SER46, ILE48, TYR49, and LEU50 residues with the IF values of 0.07, 0.22, 0.23, 0.05 and 0.17, respectively (Figure 7C). Finally, the compound CID: 51066638 (Und-GlcNAc) formed multiple interactions at LEU40, ALA41, ASN44, ILE48, LEU50, VAL51, ASN53, and THR110 of the protein with IF values of 0.18, 0.05, 0.05, 0.25, 0.6, 0.55, 0.25, and 0.1, respectively (Figure 7D).

## 4. Discussion

B lymphocytes and stratified squamous epithelium cells are the two primary target cell types of Epstein–Barr virus (EBV) [16,72]. EBV cell attachment and fusion involves several glycoproteins, including glycoprotein H (gH), gL, glycoprotein B (gB), glycoprotein 42 (gp42), and glycoprotein 350 (gp350), which are conserved throughout the herpesvirus family [29]. The gH and gL proteins integrate to form a heterodimeric gHgL complex that directly interacts with the host cell receptor on the epithelial cells. Again, the gHgL complex of EBV directly interacts with integrins αvβ5, αvβ6, αvβ8 and ephrin receptor tyrosine kinase A2 (EphA2) and hence triggers the fusion mechanism of EBV with epithelial cells [19,73]. This complex includes four distinct domains: D-I to D-IV. D-I is constructed by gL and the N terminus of gH (65 residues). Conformational change across domain I-domain II induced by the interaction between gHgL and an integrin possibly contributes to triggering membrane fusion for epithelial cells [19,74]. The gL subunit regulates the folding pattern of gH and is appointed in the specificity of EBV gB activation [74,75,76,77]. The gB is generally considered the final executor of fusion in all herpesvirus strains. The specificity of the gB activation in membrane fusion is determined by two gL residues (gL-Q54 and gL-K94), potentially through direct interactions with gB [74]. The protein gp42 is required for B cell infection and is unique to EBV. This protein binds to human leukocyte antigen (HLA) class II on the B-cell surface, contacts the gHgL complex, and facilitates viral entry into B lymphocytes [78]. 

Despite employing a sufficient workforce, the development of potential drugs without involving any dry lab approaches is costly and highly time-consuming. It takes approximately 10–15 years to bring a new drug into the market with a remarkable efficiency rate. Utilising CADD, we can strategically pick up a huge number of compounds to check their potentiality in a virtual environment. In this way, we can filter compounds with a high probability of success in the progressive stages, sparing unnecessary costs and time for the experimental part while following an efficient scientific method. Therefore, computer-aided drug design (CADD) processes have become highlighted innovations in pharmaceutical companies and research groups [79,80,81]. Structure-based drug design (SBDD), one of the two types of CADD processes, involves the evaluation of ligand interactions in the protein binding site of the 3D crystal structure of the protein [82,83]. Medicines designed using CADD processes are effective in biological environments. Therefore, CADD can be accepted as a good choice for quick and efficient drug design against pathogenic viral proteins.

In this study, we employed 628 natural compounds against pocket 2 of the target protein gL, as suggested by CASTp 3.0 and the FTmap server [50,66]. The FTmap server was considered to determine amino acids from the overall protein molecule, which can form many hydrogen bonds and non-bonded interactions. Molecular docking is a must in CADD methods to identify probable binding modes and interactions of ligands to a binding site of a specific protein according to the protein conformation and presence of amino acid residues [84,85]. We docked and screened the compounds against our target protein with a fixed grid box and considered the best four compounds according to higher binding affinity. The chosen compounds with PubChem CIDs: 4835509 (CHx-HHPD-Ac), 2870247 (Cyh-GlcNAc), 21206004 (Hep-HHPD-Ac), and 51066638 (Und-GlcNAc) showed docking scores of −5.8 kcal/mol, −5.7 kcal/mol, −5.6 kcal/mol, and −5.5 kcal/mol, respectively. In this case, although the compounds were structurally similar, it is proved that small differences in the functional groups of the compounds can produce different interactions between the compounds and the binding sites. Interpreting the interactions between the four compounds and the target protein, we found the presence of strong hydrogen and hydrophobic bonds. Though the docking scores of the four compounds were not sufficiently high, that does not eliminate them from the drug probability list. The docking scores of the candidates are much better than the control ligand. Moreover, while a lower docking score does not always mean zero potency of a compound as a drug molecule [86], a higher docking score also cannot ensure the strength of a drug candidate in in vivo or in vitro assays [87]. Therefore, further investigation was carried out to reach a significant conclusion about these four natural compounds. 

The pharmacokinetic (PK) profile investigation of lead compounds is an essential step in CADD processes. Due to poor PK profiles, many highly affinised compounds can be eliminated from the top list of probable drugs. PK observes the metabolite kinetics of the compounds in blood, tissues, cells, and organs. Absorption, distribution, metabolism, and excretion (ADME) belong to the PK properties, which regulate the activity of a drug [88]. Virtual screening can be performed to filter biologically safe and secure compounds and eliminate compounds with unpleasant properties to check the drug-likeliness of lead compounds [89]. We evaluated Lipinski’s rule of five for the four best compounds. The rule includes (i) the number of hydrogen bond donors should not exceed 5; (ii) the number of hydrogen bond acceptors should not exceed 10; (iii) the molecular mass of the compound should not exceed 500 Daltons; and (iv) the compound should possess an octal-water partition coefficient (logP) no greater than 5 [54,90]. Molecular weight affects the permeability of the drug through the biological membranes. The ability of a compound to cross lipid bilayer membranes of the human body depends on the number of hydrogen bond donors and acceptors. LogP value, sectioned under lipophilicity, affects the absorption of the compounds in the body; lower logP mediates higher absorption. We also measured the number of rotatable bonds, water solubility (LogS), GI absorption, Caco2 permeability, BBB permeability, P-glycoprotein (P-gp) substrate and inhibitor, and synthetic accessibility of the best four compounds chosen by molecular docking. The oral bioavailability of a drug relies on the number of rotatable bonds in the molecule. The solubility of the drug compounds depends on the LogS value, which should always possess a lower value [53]. According to the LogS (ESOL) scale, solubility classes are insoluble < −10 < poorly soluble < −6 < moderately soluble < −4 < soluble < very soluble < 0 < highly soluble [91]. Human epithelial colorectal adenocarcinoma cells constitute the Caco-2 cell line. Caco-2 permeability is measured to investigate the absorption of orally administered drugs through the human intestinal mucosa [55]. The BBB protects the brain from unwanted compounds and side effects. So, BBB permeability is also a significant term that minimises the side effects of drugs and enhances working capability [53]. The P-glycoprotein (P-gp), an ATP-binding cassette (ABC) transporter, acts as a biological barrier that inhibits the entry of toxins and xenobiotics and eliminates them. The P-gp substrate and inhibitor are analysed to check the compound’s ability to be transported by or to inhibit P-gp [55]. All the compounds passed most of these examinations, proving to be eligible for the human body as an efficient drug molecule.

Compounds can harm different metabolic processes inside the human body and generate abnormal activities. The doses and properties of a compound that can result in organ failures are predicted through toxicity tests. In silico toxicity testing is a popular step in rational drug design, implying CADD [92]. Toxicity reports of compounds help determine the perfect dose and the safety of a drug molecule. We determined the toxicity properties, including AMES toxicity, hepatotoxicity, carcinogenicity, oral rat acute toxicity (LD50), oral rat chronic toxicity (LOAEL), skin sensitisation, etc., for the best four compounds of our analysis. AMES toxicity describes the mutagenicity of a compound; mutagenic compounds are hazardous and can act as carcinogens. The value of LD50 describes the dose of a compound that can kill 50% of test animals; it also helps measure acute toxicity. Hepatotoxicity is essential to prevent liver injury induced by toxic molecules [55]. After analysis, we found all four molecules to be non-toxic to the human body and, hence, considered them for further analysis.

Molecular dynamics (MD) simulation is a vital computational tool to predict the durability and dynamic nature of protein–ligand complexes. It has several parameters, including RMSD, RMSF, rGyr, SASA, protein–ligand interactions, etc., that can help scientists understand the underlying interactions between the ligands and their receptors, as well as their strength, mobility, and behaviour in the presence of different solvents. This is an essential method in computer-aided drug discovery in which the stability of the complex of a selected ligand and a target protein indicates the strong binding of the ligand with the target protein. Here, we employed all of the parameters mentioned in a 100 ns MD simulation to determine the stability of our selected four best compounds with the target protein gL compared to the control ligand. We calculated the RMSD values for the chosen ligands to understand the deviation of the protein’s backbone structure upon binding with our ligands in comparison with its starting pose. Generally, an average RMSD value of a complex falling between 1 and 3 Å indicates a conformationally stable complex, whereas more significant than this range suggests changes in the conformation of the protein upon binding with the ligands, which are unfavourable [93,94,95]. Our MD simulations provided a detailed interpretation on how very similar compounds can still bind in completely different binding modes. It is of utmost importance to understand these distinctions when contemplating protein–ligand interactions in a more precise manner. In this regard, our two compounds, CID: 4835509 (CHx-HHPD-Ac) and CID: 2870247 (Cyh-GlcNAc), have average RMSD values that fall between 1 and 3 Å pointing to a stable conformation of the protein gL upon binding with these ligands separately. Moreover, the average RMSD values for both of these ligands indicate better results than the average RMSD value for the control ligand, which is another factor in considering them as a good drug candidate against EBV. However, the other two selected compounds, CID: 21206004 (Hep-HHPD-Ac) and CID: 51066638 (Und-GlcNAc), have average RMSD values greater than 3 Å, and their results are also not satisfactory compared to the control result. Furthermore, we applied the RMSF parameter during the MD simulation and estimated the average RMSF values for the four compounds and control. The RMSF values provided us with the fluctuations of protein residues upon binding with the specific ligands. In our study, CID: 4835509 (CHx-HHPD-Ac) and CID: 2870247 (Cyh-GlcNAc) compounds had the lowest average RMSF values and almost equal values compared to the average RMSF values of the control. These results also suggest fewer fluctuations of the residues of the gL upon binding with these compounds, predicting the stable complexes between the protein and the ligands. On the other hand, the other two compounds, CID: 21206004 (Hep-HHPD-Ac) and CID: 51066638 (Und-GlcNAc), had higher average RMSF values than the values of the control, especially CID: 51066638 (Und-GlcNAc), which showed the highest fluctuations throughout the simulation period. Furthermore, the rGyr and SASA values of the CID: 4835509 (CHx-HHPD-Ac) and CID: 2870247 (Cyh-GlcNAc) were much better than those of the CID: 21206004 (Hep-HHPD-Ac) and CID: 51066638 (Und-GlcNAc) compounds. The SASA values of both CID: 4835509 (CHx-HHPD-Ac) and CID: 2870247 (Cyh-GlcNAc) compounds were also superior to the value of the control ligand. Considering these analyses, it could be said that the strength of the interactions between the first two compounds (CID: 4835509 (CHx-HHPD-Ac) and CID: 2870247 (Cyh-GlcNAc)) and the protein gL of EBV is comparatively high and could produce highly stable complexes. Therefore, the compounds CID: 4835509 (CHx-HHPD-Ac) and CID: 2870247 (Cyh-GlcNAc) have the highest degree of compatibility against the gL of EBV and could inhibit the EBV. On the other hand, although the two compounds CID: 21206004 (Hep-HHPD-Ac) and CID: 51066638 (Und-GlcNAc) had high docking scores against the gL protein of EBV, the MD simulation analysis showed that their complexes with the protein are considerably unstable compared to the control ligand as well as to the other two selected compounds. Therefore, they might not be able to inhibit the EPV. Nevertheless, these analyses were based on computational tools that could only predict the results. Hence, more in vitro and in vivo studies are needed to confirm the findings of this study. 

## 5. Conclusions

Glycoprotein L is shown to be a highly potent target for inhibiting EBV infection because of its multifunctionality. It is an integral part of the gHgL complex and plays a crucial role in the virus fusion of B-cells and epithelial cells. For successful attachment and fusion, different structural conformations of the gHgL complex are essential [19,73]. According to the theoretical study, the gHgL complex requires gL for its orientation and proper folding [75,76,77]. Until now, no effective drugs have been identified to target gL protein and prevent EBV. In our current study, we performed structure-based virtual screening and identified four drug-like natural compounds with PubChem CID: 4835509 (CHx-HHPD-Ac), PubChem CID: 2870247 (Cyh-GlcNAc), PubChem CID: 21206004 (Hep-HHPD-Ac), and PubChem CID: 51066638 (Und-GlcNAc) having higher binding affinity ranging from −5.6 to −5.8 kcal/mol towards gL. However, the MD simulation results suggested that the CID: 4835509 (CHx-HHPD-Ac) and CID: 2870247 (Cyh-GlcNAc) compounds formed more stable complexes with the gL protein than the others. These results indicate that CID: 4835509 (CHx-HHPD-Ac) and CID: 2870247 (Cyh-GlcNAc) might be the better drug candidates against the EBV and can inhibit the EBV infection. Additionally, all four compounds showed harmless ADMET properties. The binding affinities and interaction characteristics analysed in our study support that these two compounds had the potential to inhibit the function of gL. They may block the fusion process of EBV and may contribute to developing and optimising new efficient EBV drugs. However, further research is needed to confirm the findings in the laboratory settings.

## Figures and Tables

**Table 1:** Grid box measurements with grid centre coordinates in x, y, and z axes and grid box size in x, y, and z dimensions.

	Axis	Value
**Center**	x	128.07503759 Å
	y	171.475486746 Å
	z	182.924808396 Å
**Size**	x	18.5380735663 Å
	y	16.2103040775 Å
	z	19.3724455668 Å

*Note: Å = Angstrom*.

**Table 2:** Amino acid (AA) residues of the assumed active site, pocket 2. The amino acids written in bold font are among the top 10 amino acids, as per the FTmap result.

Pocket ID	AA Sequence	AA Residue
2	**48**	**ILE**
2	**50**	**LEU**
2	**85**	**VAL**
2	86	VAL
2	**89**	**PHE**
2	107	LEU
2	111	LEU

Note: The control ligand of gL showed interaction with the amino acids in bold.

**Table 3:** The binding affinities of the top four drug candidates and the NAG (control ligand) with their compound identities, chemical names, molecular formula, and two-dimensional (2D) structures. The 2D structures were prepared using ACD/ChemSketch 2.0 software [68].

No.	Compound PubChem CID	Compound Name	Molecular Formula	2D Structure	Docking Score (Kcal/mol)
1	24139	Acetylglucosamine (2-Acetamido-2-deoxy-beta-D-glucopyranose) (NAG)	C_8_H_15_NO_6_	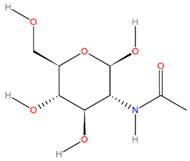	−3.9
2	4835509	N-(6-Cyclohexyloxy-8-hydroxy-2,2-dimethyl-4,4a,6,7,8,8a-hexahydropyrano[3,2-d][1,3]dioxin-7-yl)acetamide (CHx-HHPD-Ac)	C_17_H_29_NO_6_	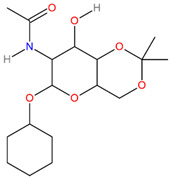	−5.8
3	2870247	Cyclohexyl 2-(acetylamino)-2-deoxyhexopyranoside (Cyh-GlcNAc)	C_14_H_25_NO_6_	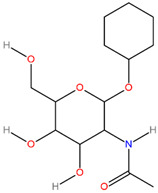	−5.7
4	21206004	N-[(4Ar,6R,7R,8R,8aS)-6-heptoxy-8-hydroxy-2,2-dimethyl-4,4a,6,7,8,8a-hexahydropyrano[3,2-d][1,3]dioxin-7-yl]acetamide (Hep-HHPD-Ac)	C_18_H_33_NO_6_	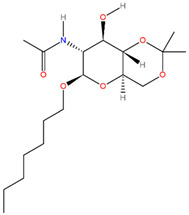	−5.6
5	51066638	Undecyl 2-acetamido-2-deoxy-B-D-glucopyranoside (Und-GlcNAc)	C_19_H_37_NO_6_	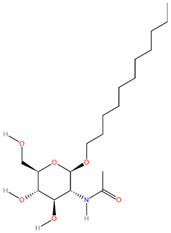	−5.5

**Table 4:** Analysis of binding interactions between the top four drug candidates and the control ligand and glycoprotein L with their binding amino acid residues, binding distance, bond category, and bond type.

PubChem CID	Residues	Distance	Bond Category	Bond Type
24139 (NAG)	ILE48	1.85	Hydrogen	Conventional Hydrogen
	ILE48	2.44	Hydrogen	Conventional Hydrogen
	LEU50	1.97	Hydrogen	Conventional Hydrogen
	LEU50	2.19	Hydrogen	Conventional Hydrogen
4835509 (CHx-HHPD-Ac)	SER46	2.45	Hydrogen	Conventional Hydrogen
	ASP47	2.87	Hydrogen	Conventional Hydrogen
	ILE48	2.8	Hydrogen	Conventional Hydrogen
	ILE48	5.23	Hydrophobic	Alkyl
	PHE89	4.43	Hydrophobic	Pi-Alkyl
2870247 (Cyh-GlcNAc)	SER46	2.95	Hydrogen	Conventional Hydrogen
	ASP47	2.77	Hydrogen	Conventional Hydrogen
	ILE48	2.32	Hydrogen	Conventional Hydrogen
	ILE48	5.3	Hydrophobic	Alkyl
	PHE89	3.62	Hydrophobic	Pi-Sigma
21206004 (Hep-HHPD-Ac)	ILE48	2.5	Hydrogen	Conventional Hydrogen
	ILE48	1.91	Hydrogen	Conventional Hydrogen
	ILE48	2.1	Hydrogen	Conventional Hydrogen
	ILE48	5.38	Hydrophobic	Alkyl
	PHE89	3.82	Hydrophobic	Pi-Alkyl
	PHE89	4.73	Hydrophobic	Pi-Alkyl
	LEU93	3.97	Hydrophobic	Alkyl
	LEU107	5.47	Hydrophobic	Alkyl
	LEU111	5.42	Hydrophobic	Alkyl
51066638 (Und-GlcNAc)	SER46	1.94	Hydrogen	Conventional Hydrogen
	ASP47	2.38	Hydrogen	Conventional Hydrogen
	ILE48	1.97	Hydrogen	Conventional Hydrogen
	ILE48	4.22	Hydrophobic	Alkyl
	LEU50	4.8	Hydrophobic	Alkyl
	PHE89	4.21	Hydrophobic	Pi-Alkyl
	PHE89	5.43	Hydrophobic	Pi-Alkyl
	PHE89	3.75	Hydrophobic	Pi-Alkyl
	LEU93	5.38	Hydrophobic	Alkyl
	LEU107	5.05	Hydrophobic	Alkyl
	LEU107	5.04	Hydrophobic	Alkyl
	LEU111	5.02	Hydrophobic	Alkyl

**Table 5:** ADME analysis results, including physicochemical properties, lipophilicity, water solubility, gastrointestinal (GI) absorption, Caco2 permeability, blood–brain barrier (BBB) permeability, etc. properties for the top four drug candidates.

Properties		CID: 4835509	CID: 2870247	CID: 21206004	CID: 51066638
Physico-chemical properties	MW (g/mol)	343.42 g/mol	303.35 g/mol	359.46 g/mol	375.50 g/mol
	Heavy atoms	24	21	25	26
	Arom. heavy atoms	0	0	0	0
	Rotatable bonds	4	5	9	14
	H-bond acceptors	6	6	6	6
	H-bond donors	2	4	2	4
	TPSA (Å)	86.25	108.25	86.25	108.25
Lipophilicity	Log P_o/w_	1.15	−0.34	1.87	2.07
Water solubility	Log S (ESOL)	−2.19	−0.99	−2.63	−3.18
Pharmacokinetics	GI absorption	High	High	High	High
Caco2 permeability	log Papp in 10^−6^ cm/s	0.9	0.025	0.89	−0.06
BBB permeability	Yes/No	No	No	No	No
P-glycoprotein substrate	Yes/No	No	No	Yes	No
P-glycoprotein inhibitor	Yes/No	No	No	No	No
Drug likeness	Lipinski, Violation	Yes; 0 violation	Yes; 0 violation	Yes; 0 violation	Yes; 0 violation
Medi. Chemistry	Synth. accessibility	4.85	4.49	5.04	5.14

**Table 6:** List of toxicological parameters such as AMES toxicity, hepatotoxicity, carcinogenicity, hERG inhibition, oral rat acute toxicity (LD50), oral rat chronic toxicity (LOAEL), etc., for the top four drug candidates.

Endpoint	Target	CID: 4835509	CID: 2870247	CID: 21206004	CID: 51066638
AMES toxicity (Yes/No)	Yes/No	No	No	No	No
Hepatotoxicity (Yes/No)	Yes/No	No	No	No	No
Carcinogenicity	Yes/No	No	No	No	No
hERG inhibition	Yes/No	No	No	No	No
Oral rat acute toxicity (LD50)	mg/kg	2.613	2.082	2.428	1.999
Oral rat chronic toxicity (LOAEL)	log mg/kg_bw/day	1.876	2.549	2.105	2.818
Androgen receptor binding	Yes/No	No	No	No	No
Skin sensitisation	Yes/No	No	No	No	No
*T.Pyriformis* toxicity	log ug/L	0.242	0.285	0.278	0.285
Minnow toxicity	log mM	3.614	3.687	2.259	1.766
Honey bee toxicity	Yes/No	Yes	No	Yes	No
Fish aquatic toxicity	Yes/No	Yes	No	Yes	No

## Data Availability

The original contributions presented in the study are included in the article/Supplementary Materials, further inquiries can be directed to the corresponding authors.

## Supplementary Materials

The following supporting information can be downloaded at: https://www.mdpi.com/article/10.3390/pathogens13110928/s1, Supplementary Table S1: The list of top 10 pockets on our target protein glycoprotein L along with their amino acid residues as predicted by the CASTp 3.0 server, Supplementary Table S2: List of the top 10 amino acids in glycoprotein L contributing to hydrogen-bonded and non-bonded interactions according to the FTmap server.
