# Supplementary material for: An In Silico Approach to Discover Efficient Natural Inhibitors to Tie Up Epstein–Barr Virus Infection
Source: Pathogens. 2024 Oct 25;13(11):928. doi: 10.3390/pathogens13110928 (PMC11597430; doi:10.3390/pathogens13110928)
Supplement: Supplementary file 1 [file pathogens-13-00928-s001.zip › pathogens-3238758-supplementary.pdf]

Supplementary Table S1: The list of top 10 pockets on our target protein glycoprotein L along with their amino acid residues as predicted by the CASTp 3.0 server. The pocket details written in bold font correspond to our chosen binding site. All the areas or volumes depicted here are solvent-accessible (SA) surface area/volume and in the unit of angstrom (Å).

Pocket ID	Area (SA)	Volume (SA)	Amino Acid Residues
1	102.578	37.074	PRO27, CYS28, CYS29, TYR49, VAL51, THR55, CYS56, LEU61, ALA62, SER63, SER77, ARG78, CYS79
**2**	**41.519**	**24.904**	**ILE48, LEU50, VAL85, VAL86, PHE89, LEU107, LEU111**
3	67.333	19.224	VAL51, SER52, ASN53, GLY58, PHE59, SER60, LEU61, ASN81, LEU83, PHE118, SER119, LEU123
4	18.539	4.622	LEU40, ASN44, ILE45, LEU64, ASN65, SER66, ILE76, ARG78
5	28.639	4.510	LEU50, VAL51, SER60, LEU61, GLY82, VAL86, LEU111, LEU114, PHE118
6	16.290	4.010	CYS29, HIS30, PHE59, LEU61, LEU128, ARG130, TYR131
7	20.100	3.271	ASP57, GLY58, SER119, VAL120, ASP122, LEU123, ALA126
8	12.805	2.456	LEU83, TYR115, PHE118, VAL120
9	2.174	0.120	SER60, LEU83, VAL46, TYR115, PHE118
10	0.358	0.009	SER52, ASN53, CYS56, PHE59

Supplementary Table S2: List of the top 10 amino acids in glycoprotein L contributing to hydrogen-bonded and non-bonded interactions according to the FTmap server. The bold amino acids are found in pocket 2 of glycoprotein L, as identified by the CASTp 3.0 server.

SL	H-bonded interaction	Non-bonded interaction
1	SER60	**ILE48**
2	VAL31	HIS30
3	HIS30	**PHE89**
4	**ILE48**	PHE59
5	LEU128	**LEU50**
6	GLY82	LEU42
7	ASN81	VAL31
8	CYS29	**VAL85**
9	**LEU50**	PHE88
10	LEU42	SER60
